# Transcript Regulation of the Recoded Archaeal α-l-Fucosidase In Vivo

**DOI:** 10.3390/molecules26071861

**Published:** 2021-03-25

**Authors:** Federica De Lise, Roberta Iacono, Andrea Strazzulli, Rosa Giglio, Nicola Curci, Luisa Maurelli, Rosario Avino, Antonio Carandente, Stefano Caliro, Alessandra Tortora, Fabio Lorenzini, Paola Di Donato, Marco Moracci, Beatrice Cobucci-Ponzano

**Affiliations:** 1 Institute of Biosciences and BioResources, National Research Council of Italy, Via P. Castellino 111, 80131 Naples, Italy; federica.delise@ibbr.cnr.it (F.D.L.); roberta.iacono@unina.it (R.I.); Rosa.Giglio@savioindustrial.it (R.G.); nicola.curci@unina.it (N.C.); luisa.maurelli@ibbr.cnr.it (L.M.); marco.moracci@unina.it (M.M.); 2 Department of Biology, University of Naples “Federico II”, Complesso Universitario Di Monte S. Angelo, Via Cupa Nuova Cinthia 21, 80126 Naples, Italy; andrea.strazzulli@unina.it; 3 Task Force on Microbiome Studies, University of Naples Federico II, 80134 Naples, Italy; 4 National Institute of Geophysics and Volcanology, Via Diocleziano, 328, 80125 Naples, Italy; rosario.avino@ingv.it (R.A.); antonio.carandente@ingv.it (A.C.); stefano.caliro@ingv.it (S.C.); 5 Kayser Italia Srl., Via di Popogna, 501, 57128 Livorno, Italy; a.tortora@kayser.it (A.T.); f.lorenzini@kayser.it (F.L.); 6 Institute of Biomolecular Chemistry, National Research Council of Italy, Via Campi Flegrei 34, 80078 Pozzuoli, Italy; pdidonato@uniparthenope.it; 7 Department of Science and Technology, University of Naples “Parthenope”, Centro Direzionale Isola C4, 80143 Naples, Italy

**Keywords:** *Archaea*, extremophiles, recoding, programmed frameshifting, limits of life

## Abstract

Genetic decoding is flexible, due to programmed deviation of the ribosomes from standard translational rules, globally termed “recoding”. In *Archaea*, recoding has been unequivocally determined only for termination codon readthrough events that regulate the incorporation of the unusual amino acids selenocysteine and pyrrolysine, and for −1 programmed frameshifting that allow the expression of a fully functional α-l-fucosidase in the crenarchaeon *Saccharolobus solfataricus*, in which several functional interrupted genes have been identified. Increasing evidence suggests that the flexibility of the genetic code decoding could provide an evolutionary advantage in extreme conditions, therefore, the identification and study of interrupted genes in extremophilic Archaea could be important from an astrobiological point of view, providing new information on the origin and evolution of the genetic code and on the limits of life on Earth. In order to shed some light on the mechanism of programmed −1 frameshifting in Archaea, here we report, for the first time, on the analysis of the transcription of this recoded archaeal α-l-fucosidase and of its full-length mutant in different growth conditions in vivo. We found that only the wild type mRNA significantly increased in *S. solfataricus* after cold shock and in cells grown in minimal medium containing hydrolyzed xyloglucan as carbon source. Our results indicated that the increased level of *fucA* mRNA cannot be explained by transcript up-regulation alone. A different mechanism related to translation efficiency is discussed.

## 1. Introduction

The decoding of genetic information into polypeptides is a dynamic mechanism in which the standard rules of decoding can be altered in special cases. In fact, in particular genes, signals encoded in the mRNA reprogram the ribosome to read the message in an alternative way, a phenomenon called translational recoding [1]. Translational recoding has been identified in organisms from all three domains of life and in viruses, and an updated list of the genes regulated by this mechanism can be found in the Recode^2^ database [2]. Recoding has crucial roles in the regulation of gene expression and the most common events are stop codon readthrough and programmed frameshifting (PRF) [1,3,4,5,6,7]. In stop codon readthrough a stop codon is decoded as a sense codon by a near-cognate tRNA. In addition to readthrough by near-cognate aa-tRNAs, stop codons can be recorded by the specialized tRNAs with an anticodon that is complementary to the stop codon, such as tRNA^Pyl^ or tRNA^Sec^, encoding for the unusual amino acids pyrrolysine [8] and selenocysteine [9]. Specific stimulatory elements downstream to the stop codon regulate this process. Events of stop codon readthrough have been reported in all the three domains of life [6]; in fact, a recent study showed that vascular endothelial growth factor-A mRNA in mammalian endothelial cells undergoes programmed translational readthrough generating an isoform containing a C-terminus extension [10]. Moreover, recoding is of interest for biomedical applications. Recent studies revealed that the premature termination codons suppression by specific drugs named “readthrough agents” may play a role in the clinical treatment of genetic diseases caused by nonsense mutations such as cystic fibrosis and Duchenne muscular dystrophy [11]. In PRF, ribosomes are induced to shift to an alternative, overlapping reading frame 1 nt 3′-wards (+1 frameshifting) or 5′-wards (−1 frameshifting) of the mRNA. The frequency of this process varies in different genes where it is under the control of sophisticated mechanisms [1]. The PRF has been studied extensively in viruses, retrotransposons and insertion elements for which many cases are documented [12,13]. Among cellular genes, where this phenomenon is less common, the best studied is the Antizyme expressed by a +1 PRF, from yeast and protists up to humans, that functions both as a sensor of the polyamine levels and as an effector of a self-regulating circuit [14].

**Figure 1:**
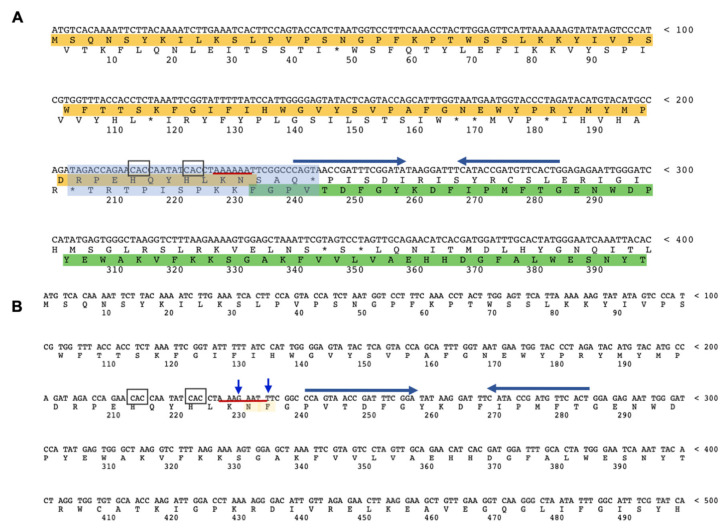
The α-l-fucosidase gene. (**A**) *fucA* gene sequence. The N-terminal SSO11867 ORF (highlighted in yellow) is in the zero frame, the C-terminal SSO3060 ORF (highlighted in green), for which only a fragment is shown, is in the −1 frame. The 40 bp region of overlap between the two ORFs is highlighted with a light blue rectangle. The slippery heptameric sequence is underlined with a red line; the rare codons are boxed, and the arrows indicate the stems of the putative mRNA secondary structure. (**B**) *framefucA* mutant gene (only a fragment is shown). The blue arrows indicate the mutated nucleotides in the slippery sequence, the insertion allowing to restore a single frame between the two ORFs (involved amino acids highlighted in yellow).

In Archaea, recoding, which was deeply studied only recently, was unequivocally demonstrated only for termination codon readthrough events that regulate the incorporation of the unusual amino acids selenocysteine and pyrrolysine [8,15], and −1 PRF that allow the expression of a fully functional α-l-fucosidase in the crenarchaeon *Saccharolobus solfataricus* [16,17,18,19,20,21]. This gene, named *fucA*, is organized in two open reading frames (ORFs) SSO11867 and SSO3060 of 81 and 426 amino acids, respectively, which are separated by a −1 frameshifting in a 40 bases overlap (Figure 1A). The analysis of the region of overlap between the two ORFs showed the characteristic features of the genes expressed by −1 PRF, including a heptanucleotide A-AAA-AAT (codons are shown in the zero frame) named slippery sequence, where the −1 PRF of the ribosome takes place, flanked by two rare CAC codons in tandem, and a putative stem–loop secondary structure (Figure 1A) resembling, respectively, the bacterial Shine–Dalgarno-like sites and stem–loops/hairpins, both with the function to slow down the translating ribosomes and promote −1 PRF. Remarkably, we demonstrated that a full-length mutant of gene, named *framefucA*, obtained by inserting specific site-directed mutations in the *fucA* gene in the positions that were predicted to generate by −1 PRF a complete polypeptide (Figure 1B) led to a functional enzyme α-l-fucosidase, named Ssα-fuc, of 495 amino acids, which resulted in it being thermophilic, thermostable, and having an unusual nonameric structure [16,17,18,19,22]. In addition, we showed that *fucA* is expressed by −1 PRF in both *E. coli* and *S. solfataricus* demonstrating, for the first time, that this kind of recoding is present in Archaea [20]. To date, only 8 archaeal α-l-fucosidases are reported and that from *S. solfataricus* is the only one characterized. It is interesting to note that the *S. solfataricus* strains P2 and 98/2, although isolated in the Pisciarelli solfataric field in Italy and in the Yellowstone National Park, respectively, shows the same interruption, suggesting a conserved regulating mechanism for this gene organization. More recently, −1 frameshifting also appears to be used by the siphoviruses tailed virus 1 (HVTV-1) and three viruses (HCTV-1, 2 and 5) that infect halophilic archaea, although the one used by the haloarchaealmyovirus tailed virus 2 (HSTV-2) is likely +1 frameshifting [23,24]. In addition, frameshifting is likely involved in the synthesis of magnesium chelatase from the archaea *Methanocaldococcus* and *Methanococcus* [25]. However, no detailed studies on the regulatory mechanism of these genes are reported. In Archaea, several functional interrupted genes have been identified in *S. solfataricus* [26]. Increasing evidence suggests that the flexibility of the genetic code decoding is a trait selected during evolution that may increase microbial fitness under certain conditions [27]. This could be particularly relevant in extreme environments, which, contrary to common believe, are not immutable but subjected to sudden changes that greatly, and temporarily, modify the chemical-physical parameters and to which microorganisms must adapt. For these reasons the identification and study of interrupted genes in extremophilic Archaea are important from an astrobiological point of view and can provide new information on the origin and evolution of the genetic code and on the limits of life on Earth and beyond.

Unfortunately, the study of recoding is still very limited in extremophilic Archaea due to the difficulties of growing them in laboratory, the lack of reliable tools for gene manipulation, and for the limited knowledge of the physiology of these organisms in vivo. *fucA* is the only archaeal gene that has been demonstrated to be expressed by −1 PRF and, since it encodes for an enzyme that can be easily assayed in-vitro, it is an ideal molecular system to study −1PRF in vivo. In particular, understanding why *fucA*, a gene presumably involved in carbohydrate metabolism, is expressed by recoding and if its expression is regulated by specific growth conditions or metabolites, could help to shed some light on how this mechanism evolved and is regulated in Archaea. Here, to test if the expression of *fucA* is regulated in vivo, and in an effort of identifying external effectors involved in its expression, we analyzed its transcription and the enzymatic activity of the α-l-fucosidase in different growth conditions. In particular, we compared the wild type strain, in which the expression of *fucA* is controlled by −1 PRF, with a mutant strain in which we inserted the full-length gene and whose expression is therefore not translationally regulated by −1PRF. We report here that, in some conditions, the mRNA level of the wild type transcript increased up to 10-fold, while the level of the full-length transcript remained almost unchanged. The possibility that the increase in *fucA* transcript in the wild type is due to improved translation efficiency rather than transcription up-regulation is discussed. 

## 2. Results

### 2.1. Knocked-Out and Full-Length fucA S. solfataricus Strains

**Figure 2:**
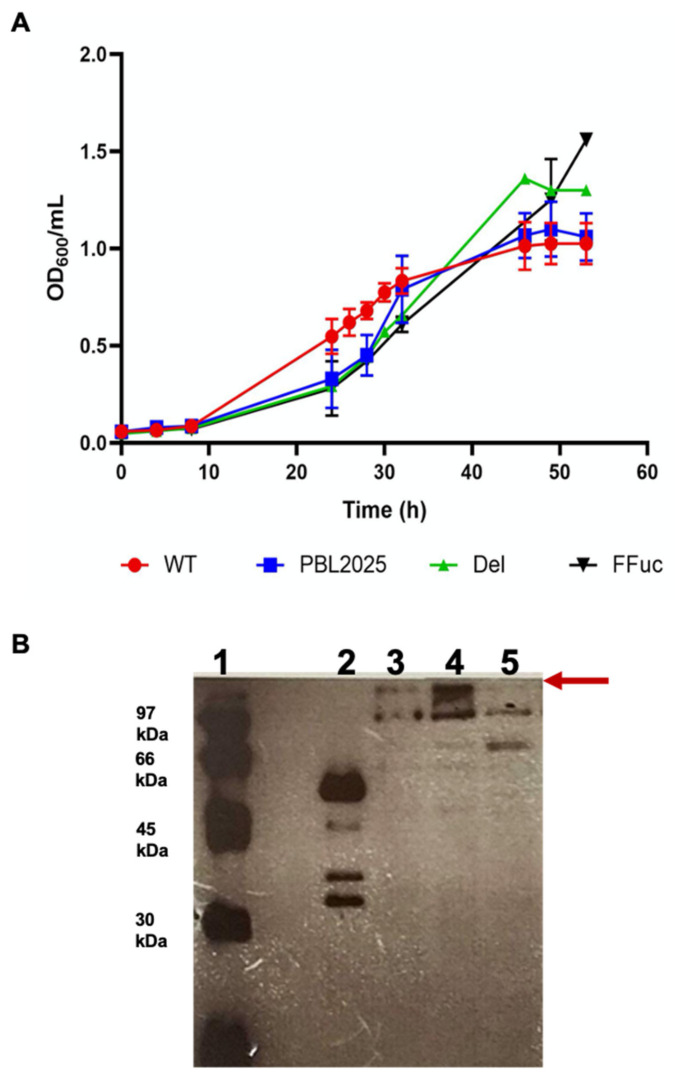
*S. solfataricus* wild type and mutant strains. (**A**) Representative growth curves *of S. solfataricus* wild type (WT), PBL2025, Del and FFuc strains (red, blue, green and black lines, respectively) in YCS medium at 80 °C. Error bars reported represent standard deviations. (**B**) Western blot analysis, performed with anti-α-l-fucosidase antibody, on cellular extracts of *WT*, FFuc and Del mutants. Lane 1: ECL markers (97-66-45-30 kDa), lane 2: α-l-fucosidase 0.1 mg/mL; the additional bands observed are due to partial proteolysis as already reported [22]; lane 3: wild type extract; lane 4: FFuc extract; lane 5: Del extract. The apparent high molecular bands corresponding to the α-fucosidase in vivo are indicated by a red arrow.

The essentiality of the α-l-fucosidase gene in *S. solfataricus* was analyzed by preparing a deletion mutant PBL2025:ΔSSO3060-SSO11867 (Del) and comparing it with the parental wild type (*WT*) and PBL2025 (a *S. solfataricus* strain deleted of 50 genes, from ORFs SSO3004 to SSO3050, many of which encode for carbohydrate-active enzymes) strains in standard conditions. To prepare the Del mutant, 444 nucleotides, internal to the gene, from position 192 to position 638, were deleted. The strain has been controlled by PCR. The deletion resulted in the introduction of a stop codon after 213 nucleotides from the ATG of the first ORF SSO11867. This could only result in the translation of a polypeptide of 71 amino acids, ruling out the translation of a full-length protein and a functional enzyme. As reported in Figure 2A, the Del strain is viable, clearly indicating that *fucA* is not an essential gene for *S. solfataricus* grown in this condition. In order to analyse the effect of the presence of a full-length gene, not regulated by −1PRF, we prepared a mutant strain of *S. solfataricus* in which the interrupted wild type *fucA* gene was substituted with the full-length mutant *framefucA* (FFuc strain). This mutant has been prepared by replacing the same 444 nucleotide sequence of the wild type, as described above, with those of the *framefucA* full-length mutant (Figure 1B). Therefore, in all these mutants the possible transcription regulatory signals remained unchanged. By comparing the growth curves, we observed that all strains were viable, but the mutants had a slightly longer latency phase than *WT* (Figure 2A). Western Blot analysis (Figure 2B) performed on the cellular extracts of both wild type and the two mutants, using antibodies against α-l-fucosidase, confirmed that the higher molecular band revealed in *WT* cellular extracts corresponded to the oligomeric form of the α-l-fucosidase as previously reported [20,22]. As expected, a more intense signal was observed in the full-length mutant FFuc strain. The high apparent molecular weight of the bands in lanes 3–4 is due to the higher stability of the nonameric structure of the enzyme in the *S. solfataricus* extract if compared to the purified recombinant α-l-fucosidase, as already reported [20,22]. As reported previously [20], unspecific signals for bands lower than 97 kDa and a very faint band visible in the Del lane, were detected. The band observed at about 70 kDa in the Del mutant probably represent a multimer of the 71 amino acids polypeptide.

**Figure 3:**
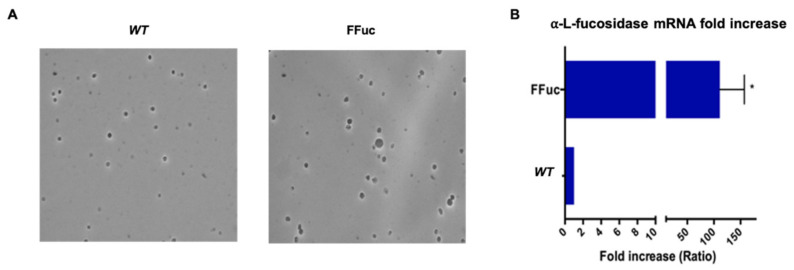
Comparison of *S. solfataricus* wild type and FFuc strains. (**A**) Contrast phase microscopy of of *WT* and FFuc cell cultures harvested in late exponential phase; (**B**) Transcriptional analysis of *fucA* and *framefucA* mRNA in *S. solfataricus WT* and FFuc strains, respectively, analyzed by real-time PCR. The amount of *fucA* and *framefucA* mRNA were determined in cultured cells and normalized against 16S (internal control). The amount of mRNA in the *WT* was set as 1 and that present in FFuc was normalized accordingly. Data are representative of three measurements and are expressed as the mean ± SD. Statistical significance was performed using the two-tailed paired Student’s *t* test (* *p* < 0.05).

*WT* and FFuc strains grown in YCS were analyzed after 48 h of growth (late exponential phase, FFuc 1.0, *WT* 0.9 OD_600_) by contrast phase microscopy and no clear morphological differences could be observed between the two strains (Figure 3A). To get more insights on the possible differences between the two strains at molecular level, we analyzed and compared the transcript level of *fucA* and *framefucA,* and the enzymatic units of α-l-fucosidase in *WT* and FFuc strains. In particular, the activity of the enzyme is a convenient indication of the expression of a full-length polypeptide. Interestingly, in the FFuc mutant the α-l-fucosidase activity was 8-fold higher than in the *WT* strain (18.0 vs. 2.3 mU/mg) in standard conditions, as expected from a full-length gene. The mRNAs from *WT* and FFuc strains, grown in standard conditions and recovered in the late exponential phase as above, were analyzed by Real-time PCR. The ratio between wild type and mutant mRNA was the result of three different measures and the transcript level of each sample was normalized by using 16S rRNA specific oligonucleotides. The analyses showed that the amount of mRNA extracted from the FFuc mutant (bearing the full-length gene) is 100-fold higher than the mRNA extracted from the wild type strain in which the gene was interrupted (Figure 3B). It is well known that mRNAs with a premature stop codon (PTC) are recognized and targeted for degradation [28,29,30]. Thus, this result suggested that the presence of the −1 frameshift could acted as a *cis-acting* mRNA destabilizing element, targeting part of the wild type mRNA for degradation. By contrast, the full-length mRNA remained stable.

### 2.2. Environmental Conditions Variation in Pisciarelli Solfatara

**Figure 4:**
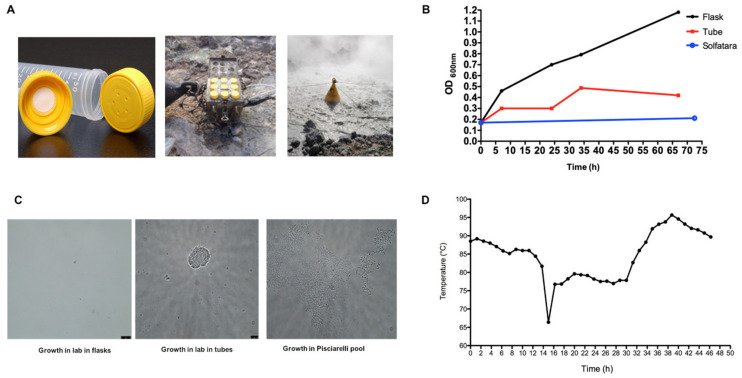
Growth of *S. solfataricus* in solfatara Pisciarelli. (**A**) Device for the *S. solfataricus* growth in the Pisciarelli pool; (**B**) growth curves of *S. solfataricus WT* in YCS medium, in lab (flasks and plastic tubes at 80 °C, black and red, respectively) and in Pisciarelli pool in plastic tubes (blue); (**C**) contrast phase microscopy of cultures in lab, in flasks and plastic tubes, respectively and in Pisciarelli pool in plastic tubes; (**D**) fluctuation temperatures measured in Pisciarelli pool during *S. solfataricus* incubation.

It has already been well documented that PRF increases the coding potential of the genome, it is often used to ensure a defined stoichiometry of protein products, to expand the variability of cellular proteomes or adapt to changing environments [27]. The special living conditions of Archaea, in particular of *S. solfataricus* growing at *T* = 80 °C and pH 3.0–5.0 in geothermal sites, make this organism an interesting model system to study whether translational recoding is involved in adaptations to extreme environment, stress responses and changing growth conditions. On the other hand, to predict which specific elements could be involved in the regulation of *fucA* is not easy. To evaluate the effect of the natural environment we exposed the wild type laboratory strain to a water-mud pool in the Pisciarelli solfataric field (Figure 6), where it was originally isolated [31]. The *S. solfataricus* grown in the laboratory up to 0.17 OD_600nm_ was divided into 3 groups of sample cultures incubated as follows: (i) in the Pisciarelli solfatara using tubes capped with a 0.22 µm filter, which allowed the exchange of trace elements present in the solfatara water, but not of microorganisms and sediments; (ii) in the lab in the same tubes, but in a shaker at 80 °C; (iii) in the lab in a standard laboratory flask at 80 °C, in a shaker. To incubate the samples in the Pisciarelli solfatara, a device, specially designed and built in collaboration with Kayser Italia, was used. The device was anchored and kept floating by a buoy (Figure 4A). The temperature of the Pisciarelli solfatara pool was monitored using a thermometer inserted in the incubation device. The OD_600nm_ were monitored in the laboratory controls for the same incubation time as in Pisciarelli, and for the following three days. As shown in Figure 4B, the growth of the controls in the tubes was slower than in the flask, probably due to reduced oxygenation. When the control culture in the flask exceeded 1.0 OD_600nm_, the tubes were recovered from the solfataric pool. At the end of incubation, the following OD_600nm_ were measured: Pisciarelli tubes 0.21, lab flask 1.18 and lab tubes 0.42. During the experiment, the temperature in Pisciarelli was rather stable, between 80 and 90 °C; however, fluctuations were also observed with a negative peak at 66 °C, and increments up to 96 °C, in response to heavy rain events and/or changing in the hydrothermal gas flux discharged by the Pisciarelli pool (Figure 4D). A morphological analysis of the three samples by contrast phase optical microscopy revealed the presence of cellular aggregates only in the samples incubated in the Pisciarelli solfatara (Figure 4C). This is similar to UV-induced stress response. In fact, it has been reported that the stress induced by UV irradiation of *S. solfataricus* induces an archaeal pili system, which mediates cellular aggregation in response to UV damage [32,33].

Unfortunately, the very low amount of proteins extracted from the sample incubated in the Pisciarelli pool (1.6 mg/mL), if compared to the control samples in the tubes (10.9 mg/mL) and in the flask (16.2 mg/mL), prevented a detailed comparative proteomic analysis of the three samples in order to evaluate *fucA* and other interrupted genes expression in these conditions. Since the incubation of samples in the solfataric field was difficult to replicate and monitor, we decided to analyse whether different growth conditions could regulate the transcription and translation of *fucA* under controlled experiments in the lab.

### 2.3. Transcript and Enzymatic Activity Levels of α-l-Fucosidase in Different Conditions

The reason why the expression of *fucA* is regulated by frameshifting in vivo is not known. Several lines of evidence allowed us to exclude that −1 PRF is used to set the ratio of two polypeptides for the α-l-fucosidase but, rather, we suggested that it is used to regulate the expression of a functional full-length product [17,18,19,20]. Thus, the natural frameshifting levels of *fucA* could likely vary depending on growth condition and physiological state, as reported for *E. coli* in which frameshifting levels increase entering in the stationary phase, presumably due to starvation and/or aa-tRNAs limitation [34,35], or in the case in which a higher α-l-fucosidase activity is requested for specific physiological reason. 

We observed that, in standard growth conditions, *fucA* produced a rare transcript [20] and expressed a low α-l-fucosidase activity. To get more insights into the possible regulation of the transcription and translation of *fucA*, the *WT* and FFuc strain were grown in different conditions and compared at molecular level by measuring transcript level, by Real-time PCR, and the α-l-fucosidase activity, by enzymatic assays.

#### 2.3.1. Cold Shock and UV Irradiation

**Figure 5:**
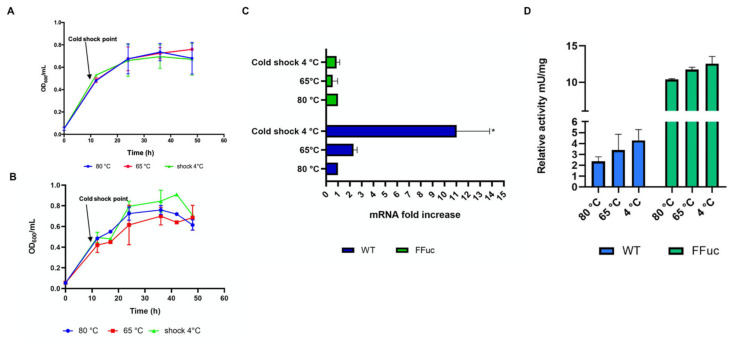
*S. solfataricus strains* response to cold-shock stress. (**A**) Growth curve of *S. solfataricus WT* strain at 80 °C (blue) and subjected to cold shock at 65 and 4 °C (red and green, respectively). Data are representative of three independent growth curves and are expressed as the mean ± SD. (**B**) Growth curve of *S. Solfataricus* FFuc strain at 80 °C (blue) and subjected to cold shock at 65 and 4 °C (red and green, respectively). (**C**) Real-time PCR of *fucA* and *framefucA* mRNA. The amount of mRNA in each strain grown at 80 °C was set to 1 and the other values were normalized accordingly. Data are representative of three measurements and are expressed as the mean ± SD. Statistical significance was performed using the two-tailed paired Student’s *t* test (* *p* < 0.05) and ANOVA Dunnett’s test (*p* < 0.0013). (**D**) Activity assays on *WT* and FFuc *S. solfataricus* cellular extracts on 4NP-α-l-fucopyranoside. Relative activity of α-l-fucosidase is expressed as mU/mg.

As observed above, in its natural environment, *S. solfataricus* may have to face sudden changes in temperature to which it must quickly respond. To evaluate the possible impact of cold shock on the *fucA* gene expression, cold shock time course experiments were carried out and growth curves of the wild type and FFuc strains were monitored up to stationary phase (Figure 5). As reported in Figure 5A,B, cells viability of both strains at 65 °C, and after cold shock at 4 °C, was not affected, and the growth curves were comparable to the control at 80 °C, with no significant differences. Instead, *fucA* mRNA showed a 10-fold increase in cold shocked cells at 4 °C, and 2-fold in cells grown at 65 °C (Figure 5B). Surprisingly, in the FFuc strain, the *framefucA* mRNA was not affected in any of the conditions tested, suggesting that the observed increase of mRNA in the *WT* strain after cold shock was not due to transcriptional up-regulation. Moreover, in *WT* cells cold shocked at 4 °C, we observed a 2-fold increase of α-l-fucosidase activity (from 2.0 to 4.2 mU/mg at 80 and 4 °C, respectively) (Figure 5D). By contrast, the α-l-fucosidase activity remained almost constant in the cellular extract of FFuc strain.

**Figure 6:**
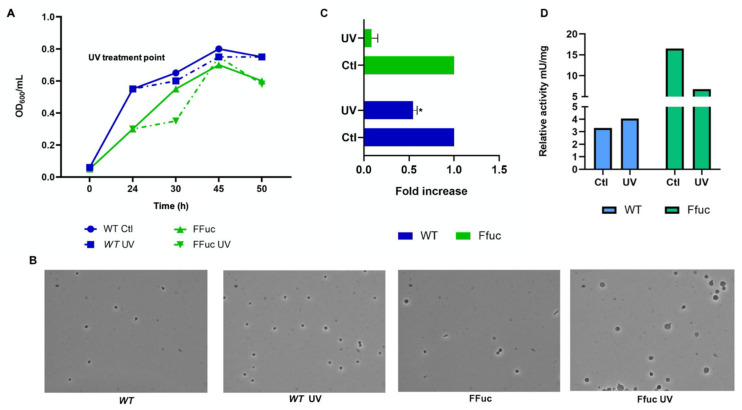
Response of *S. solfataricus* to UV irradiation. (**A**) Representative growth curve of *S. solfataricus* WT (blue lines) and FFuc (green lines) in standard conditions and after UV irradiation. (**B**) Contrast phase microscopy of cell cultures harvested after 50 h. (**C**) Real-time PCR of *fucA* and *framefucA* mRNA. The level of each mRNA was normalized against 16S (internal control). The mRNA amount of the non-treated *WT* and FFuc was set to 1 and the other values were normalized accordingly. Data are representative of three measurements and are expressed as the mean ± SD. Statistical significance was performed using the two-tailed paired Student’s *t* test. (* *p* < 0.05). (**D**) Activity assays of WT and FFuc cellular extracts on 4NP-α-l-fucopyranoside. Relative activity of α-l-fucosidase is expressed as mU/mg.

Following these results, we decided to evaluate the effect of UV irradiation, another stressor commonly used for *S. solfataricus* [36,37] on both strains. We analyzed the cells’ viability after 60 J/m^2^ UV doses of UV-C (254 nm) [38] by monitoring the growth curve of the *WT* and FFuc strains after UV irradiation (Figure 6). After UV irradiation, the strains showed the same growth rate and morphological aspect of the controls (Figure 6A,B). Real-time PCR analysis revealed that the amounts in mRNA of both *fucA* and *framefucA* were reduced after UV irradiation (2- and 10-fold respectively) (Figure 6C), confirming a transcriptional down-regulation of this gene after UV irradiation as already reported [36]. The enzymatic activity assays revealed that in the FFuc strain the decrease of the transcript well correlated with the decrease of the enzymatic activity (16.5 vs. 6.8 mU/mg, control and UV, respectively). Instead, in the *WT* strain, the enzymatic activity is comparable to that of the control (3.3 and 4.0 mU/mg, control and UV respectively), despite a 2-fold decrease of the transcript level (Figure 6D).

#### 2.3.2. Carbon Sources

**Figure 7:**
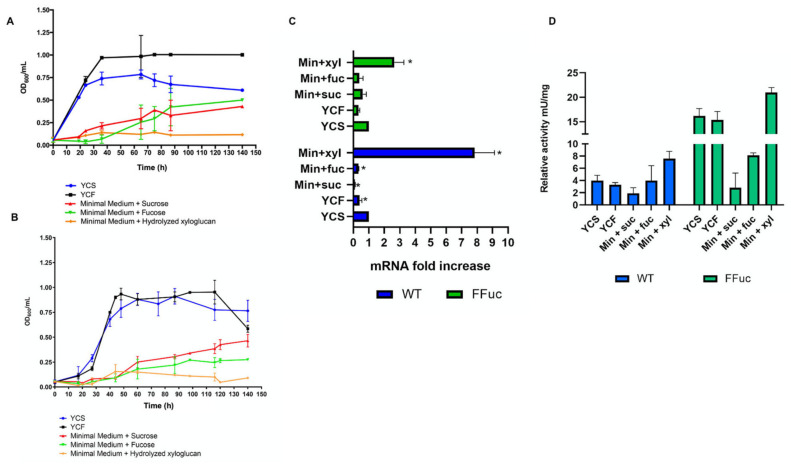
*S. solfataricus* strains growth with different carbon sources. (**A**) Representative growth curves of *S. solfataricus WT* strain in different media: YCS (blu), YCF (black), Minimal Medium + Sucrose (red), Minimal Medium + Fucose (green) and Minimal Medium + Hydrolyzed Xyloglucan (orange). Data are representative of three independent growth curves and are expressed as the mean ± SD. (**B**) Representative growth curves of *S. solfataricus FFuc* strain in different media: YCS (blu), YCF (black), Minimal Medium+ Sucrose (red), Minimal Medium + Fucose (green) and Minimal Medium + Hydrolyzed Xyloglucan (orange). (**C**) Real-time PCR of *fucA* and *framefucA* mRNA. The level of each mRNA was normalized against 16S (internal control). The mRNA amount in YCS was set to 1 and the other values were normalized accordingly. Data are representative of three measurements and are expressed as the mean ± SD. Statistical significance was performed using the two-tailed paired Student’s *t* test (* *p* < 0.05) and ANOVA Dunnett’s test (*p* < 0.0001). (**D**) Activity assays on WT and FFuc *S. solfataricus* cellular extracts on 4NP-α-l-fucopyranoside. The relative activity of α-l-fucosidase is expressed as mU/mg.

*S. solfataricus* possesses a broad capacity for the degradation of different polymeric sugars as documented by the presence of 28 glycoside hydrolases in its genome (according to http://www.cazy.org (accessed on 4 March 2021)) [39,40]. Plant material originating from woodland areas surrounding Pisciarelli solfatara [41], mainly contribute to the presence of different polysaccharides and glycoconjugates in the organism’s natural habitat. Thus, *S. solfataricus* adapted its metabolism to these environmental conditions, but relatively little is known about the function of these enzymes in vivo. However, several genes encoding for glycosyl hydrolases, namely an α-glucosidase (SSO3051), a β-glucuronidase (SSO3036), a β-xylosidase (SSO3032), and the clustered α-xylosidase (XylS) and α-glycosidase (Ssβ-gly) (SSO3022 and SSO3019, respectively), map close to *fucA* and are likely to be involved in the degradation of polysaccharides for energy metabolism [42,43,44,45,46,47,48,49]. To evaluate whether the transcription and the translation of the *fucA* gene could be affected by growing *S. solfataricus* with different carbon sources, the *WT* and FFuc strains were grown in rich and minimal media supplemented with different sugars. As shown in Figure 7A,B, both strains reached the stationary phase after 50 h in the two rich media supplemented with sucrose or fucose, respectively. By contrast, they both showed an extremely slow growth in all three minimal media supplemented with sucrose, fucose and hydrolyzed xyloglucan. Real-time PCR analysis performed on the mRNA extracted after 90 h of growth revealed a slight transcription down-regulation for both strains grown in YCF, minimal media with sucrose or fucose (Figure 7C). By contrast, interestingly, we observed a 10-fold increase of *fucA* mRNA in *WT* cells grown in minimal medium supplemented with hydrolyzed xyloglucan (Figure 7C, blue bars). A 2-fold increase of *framefucA* mRNA has been observed also in the FFuc mutant (Figure 7C, green bars). Furthermore, in cells from *WT* we found a 2-fold increase of α-l-fucosidase enzymatic units when compared to that found in YCS (from 4.0 to 7.6 mU/mg). Instead, in FFuc strain the increase is 1.3-fold (from 16.2 to 21.0 mU/mg) (Figure 7D).

## 3. Discussion

Increasing evidence suggests that the flexibility of translation may increase microbial fitness under certain conditions [27]. This could be particularly relevant in extreme environments, which are subjected to sudden environmental changes, as showed in Figure 4D, to which microorganisms must rapidly adapt. PRF is one of the forms of recoding that regulates and enriches gene expression. However, the physiological significance of PRF has been assigned only to a minority of the cellular genes while for most of them it is still uncertain [1,6,13,14,50]. The reason why *fucA* expression is regulated by −1 PRF in *S. solfataricus* is not known. The polypeptide encoded by the smaller ORF SSO11867 could never be detected by Western Blot or proteomic analyses [20,26,51] In addition, the modelling of Ssαfuc on the high-resolution crystal structure of the α-l-fucosidase from *Thermotoga maritima* showed that the N-terminal polypeptide is not an independent domain [22] and we have shown that SSO11867 includes essential catalytic residues [18], excluding the possibility that a functional α-l-fucosidase can be obtained from the C-terminal ORF SSO3060 alone. Therefore, several lines of evidence allowed us to exclude that −1 PRF is used to set the ratio of the two polypeptides, rather we suggested that this translational mechanism might be required to control the expression level of the α-l-fucosidase [20].

To get more insights into the mechanism of regulation of *fucA* in *S. solfataricus,* we compared the transcript level and the α-l-fucosidase activity of the *WT* strain with those of the FFuc strain in which *fucA*, being full-length (*framefucA*), is not under the regulation of −1 PRF.

Here, we found that the mRNA level of *framefucA* in FFuc strain is 100-fold higher than that of the *WT* in standard conditions. It is well known that faulty mRNAs with PTC are recognized and degraded by NMD [28,29,30]. These data suggested that the wild type mRNA is recognized as an mRNA containing a non-sense mutation and initiated towards a degradation pathway of NMD [30]. However, the presence of the full-length α-l-fucosidase [20] and of the α-l-fucosidase activity suggested that low level of −1 PRF occurred in standard conditions. In contrast, the mRNA of the FFuc strain, as expected for a full-length gene, is stable and efficiently translated as suggested by the higher α-l-fucosidase activity in cellular extracts of the FFuc strain.

Therefore, we decided to analyze the behavior of the two strains under different stress conditions. Indeed, this comparison could provide useful information on the regulation of *fucA* in vivo. Surprisingly, after cold shock, we observed a 10-fold increase of the wild type transcript in *WT* strain, while in FFuc the full-length transcript remained constant. We also observed an increase in the α-l-fucosidase activity of 2-fold. Since the putative regulatory transcriptional sequences of the wild type and mutant genes are the same, this suggests that the increase in wild type transcript was not due to transcriptional up-regulation. It has been proposed that recognition of a nonsense mRNA could depend on translation [52] and that mRNA depletion is a consequence of the appearance of long tracts of mRNA that are unprotected by scanning ribosomes [28], which, by binding to the mRNA, have a protective effect on its stability [53]. Thus, we explain the higher amounts of fucA mRNA with an increment of translating ribosomes, which stabilize fucA mRNA and lead to the increased transcript level revealed by Real-time PCR. These results are consistent with the proposed strategy adopted by the Sec codon to avoid detection as PTC by the NMD surveillance pathway, linking the selenoprotein synthesis to the efficiency of Sec incorporation. Under the conditions of adequate dietary selenium, when Sec-tRNASec levels are high, NMD presumably does not occur because Sec incorporation is efficiently out-competing translation termination [54,55,56]. Our data show that cold shock had an effect in vivo on the *fucA* gene, which is expressed by −1 PRF. Although both *fucA* mRNA abundance and α-l-fucosidase specific activity increase after cold shock, the variation is not comparable (10- vs. 2-fold in Figure 5C,D). Possibly, the increment rates for mRNA stabilization and protein translation is different, they depend on the −1 PRF efficiency, or α-l-fucosidase specific activity observed in these conditions was enough for the required biological effect. The involvement of α-l-fucosidase in cold shock is not clear and requires further studies that go beyond the aims of this work.

After UV irradiation, we observed a decrease in the transcript level for both wild type and mutant genes, clearly indicating that *fucA* is subjected to transcriptional down-regulation under this stress condition as already reported [36]. On the contrary, when *WT* was grown in the presence of hydrolyzed xyloglucan, we observed a 10-fold increase in the level of wild type mRNA and an increase of 2-fold in α-l-fucosidase activity. We observed also a slight (2-fold) increase in transcription and enzymatic activity (1.3-fold) in the FFuc strain. These data suggest that in these conditions transcriptional and translational up-regulation occur in both the *WT* and FFuc strains. In the full-length gene the increase in the transcript level was much lower than in the wild type gene, suggesting that, as we proposed for the cold shock experiments, the translating ribosomes stabilized the mRNA of the wild type. The increasing demand of α-l-fucosidase in the presence of xyloglucan oligosaccharides was not surprising. Several α-l-fucosidases belonging to glycoside hydrolases family GH29 in the Carbohydrate Active enZYmes database (http://www.cazy.org/ (accessed on 4 March 2021)) [39,40,41,42,43,44,45,46,47,48,49,50,51,52,53,54,55,56,57,58]. In addition, we have previously reported that in *S. solfataricus* the α-xylosidase XylS and the β-glycosidase Ssβ-gly, hydrolyzed tamarind seed xyloglucan oligosaccharides in vitro [48]. More recently, we observed that the α-l-fucosidase is able to remove the fucose residues from fucosylated xyloglucan oligosaccharides [59], suggesting that the three enzymes cooperate for the hydrolysis of xyloglucan oligosaccharides [17] like it has been suggested in *T. maritima* [49].

We propose that we observed an increase of the wild type mRNA in grown conditions in which the α-l-fucosidase is required due to the stabilizing effect of the ribosomes which translate through −1 PRF the interrupted gene. Why the α-l-fucosidase is regulated in these, or other, grown conditions it certainly deserves further study, in order to provide new information on the possible link between the mechanism of −1 PRF in Archaea and increased fitness in extreme environments.

## 4. Materials and Methods

### 4.1. Culture Media

YCS: Brock’s salt medium supplemented with yeast extract (0.1%), casamino acids (0.1%), and sucrose (0.1%) [60].

YC: Brock’s salt medium supplemented with yeast extract (0.1%) and casamino acids (0.1%) [60].

YCF: Brock’s salt medium supplemented with yeast extract (0.1%), casamino acids (0.1%), and fucose (0.1%) [60].

### 4.2. Strains and Growth Conditions

*S. solfataricus* strain P2 (DSM1617) and strain 98/2 (PBL2000) were considered indifferently as wild type strains [61].

*S. solfataricus* strain PBL2025 is a natural deletion mutant of strain 98/2, in which a fragment of about 50 kb of the chromosome containing ~50 ORFs (SSO3004-3050) is missing [62].

The mutant strains Del and FFuc were outsourced by the Gene Expression company (Gene Expression Center for Biotechnology, University of Nebraska-Lincoln, Lincoln, NE, USA). The mutants were obtained by using the PBL2025 as the parental strain. The two ORFs encoding for the α-l-fucosidase are located on a DNA region of 1487 bp. The mutant strain Del was obtained by deleting 444 bp of the *fucA* wild type gene (from position 195 to position 635), which includes the region of overlap between the two ORFs. The FFuc mutant strain was obtained by replacing the same 444 bp DNA sequence of the wild type with the DNA of the *framefucA* mutant, in which the two ORFs were carried on the same reading frame through site directed mutagenesis. The Del mutant strain has been controlled by PCR. The internal deletion of 444 bp determined the insertion of a stop codon after 71 amino acids from the ATG, preventing the translation of a full-length protein.

Unless otherwise indicated, *S. solfataricus* strains were grown at 80 °C, pH 3.5 in Brock’s salt medium supplemented with yeast extract, sucrose, and casamino acids (0.1% each) [60]. The growth of cells was monitored spectrophotometrically at 600 nm and the cells were harvested at the early stationary phase (0.7–1.0 OD) by centrifugation at 5000× *g* for 15 min at 4 °C.

### 4.3. Growth of S. solfataricus P2 in Pisciarelli Solfatara Pool

*S. solfataricus* wild type, grown up to 0.17 OD_600nm_in 100 mL of YCS in long neck flasks, was divided into 3 samples: (i) in the first sample, the culture was incubated in the Pisciarelli solfatara by using tubes having a 0.22 µm filter at the top of the cap, which allowed the exchange of trace elements present in the solfatara water but not of microorganisms and sediments; (ii) in the second sample the cells were incubated in the same tubes but in controlled conditions (in the lab); (iii) in the third sample the cells were incubated in controlled conditions (in the lab) but in a standard laboratory 250 mL flask. The temperature of the pool was monitored for the duration of the experiment using a thermometer inserted into the device. From the time of incubation in Pisciarelli and for the following three days, the OD_600nm_ have been measured in the laboratory controls. 

### 4.4. Growths in Different Carbon Sources

For each culture, *S. solfataricus WT* and FFuc strains were inoculated in long neck flasks in 100 mL of YCS. Growth rate was monitored spectrophotometrically at 600 nm. Each culture (initial OD_600_: 0.04) were incubated at 80 °C, pH 3.5 and under shaking at 160 rpm overnight (ON). Once the culture reached the early exponential phase (0.4–0.5 OD_600_), it was diluted to a value of 0.05 OD_600_ in 250 mL of fresh media and growth at 80 °C.

1. Rich medium (YCS): Brock’s salt medium supplemented with yeast extract (0.1%), casamino acids (0.1%), plus sucrose (0.1%).

2. Minimal medium + Sucrose: Brock’s salt medium supplemented with sucrose (0.1%).

3. Minimal medium + Fucose: Brock’s salt medium supplemented with fucose (0.1%).

4. Minimal medium + Hydrolyzed Xyloglucan: Brock’s salt medium supplemented with non fucosylated hydrolyzed Xyloglucan mix (0.1%).

5. Rich medium with fucose (YCF): Brock’s salt medium supplemented with yeast extract, casamino acids and fucose (0.1% each).

Growth rate was monitored spectrophotometrically at 600 nm and cultures were harvested after 90 h (late exponential phase), centrifuged at 3500× *g* for 5 min and stored at −20 °C until use.

### 4.5. Cold Shock

*S. solfataricus WT* and FFuc strains were inoculated in long neck flasks in 100 mL of YCS and incubated at 80 °C, pH 3.5 and under shaking at 160 rpm overnight (ON). Growth rate was monitored spectrophotometrically at 600 nm. Once the culture reached the logarithmic phase of growth (0.4–0.5 OD_600_), it was diluted to a value of 0.05 OD_600_ in three aliquots of 250 mL of fresh YCS medium and cultured at 80 °C up to 0.5 OD_600_; once arrived in early exponential phase, each strain culture was incubated (i) at 65 °C (ii) at 4 °C for 2 h and then again at 80 °C. As control one culture of each strain was grown at 80 °C as described above. For each culture, growth rate was monitored spectrophotometrically using a Cary 100 (Agilent, Santa Clara, CA, USA) at 600 nm and cells were harvested during the early stationary phase, at 0.8 OD_600._ Cells were centrifuged at 3500× *g* for 15 min and pellets stored at −20 °C.

### 4.6. UV Irradiation

*S. solfataricus WT* and FFuc strains were grown in 100 mL of YCS up to 0.4–0.5 OD_600_. An aliquot of 50 mL was put in ice and used for UV-irradiation. Irradiation was performed by means of a UV-C lamp (model EF-280C/FE 230-volt 50 Hz 0.34 AMPS. Spectroline, Westbury, NY, USA) set at λ = 254 nm with a power of about 6.00 W/m^2^. The UV flux at the surface of cells was measured with a UV digital radiometer (HD 2102.2. Delta OHM, Caselle di Selvazzano (PD), Italy). The experiment was carried out in sterile conditions: in particular, 5 aliquots of 10 mL were transferred to a plastic petri dish (110 mm) and irradiated with UV light for 20 s at 245 nm (corresponding to an absorbed dose of 60 J/m^2^) while shaking the culture carefully. The treated cultures were stored in the dark on ice for 30 min and then incubated at 80 °C under shaking. Cell growth was spectrophotometrically monitored, and the samples were harvested after 50 h. Cells were centrifuged at 3500× *g* for 15 min and pellets stored at −20 °C until use.

### 4.7. RNA Extraction and Real-Time PCR 

Total cellular RNA was extracted from *S. solfataricus WT* and FFuc cells, according to the Qiagen’s RNeasy mini protocol, with a DNase step treatment, at 37 °C for 20 min, when necessary. Total RNA concentrations were estimated with Qubit 4 Fluorometer using the Qubit™ RNA HS Assay Kit (Thermo Fisher, Waltham, MA, USA) and RNA quality was independently assessed by visualization on a 1.5% agarose (wt/vol) gels. cDNA was synthesized from 300–600 ng of total RNA from each sample using SuperScript™ IV VILO™ Master Mix reverse transcriptase (Thermo Fisher, Waltham, MA, USA) in a 25 μL reaction. The reverse transcription reactions were performed according to Thermo Fisher protocol. In particular, for each a reaction mixture containing 4 µL of RT reaction mix and 300–600 ng of RNA was prepared as a control the same mixture without reverse transcriptase was used. The program used was as follows:10 min at 25 °C, 10 min at 50 °C, 5 min at 85 °C.

The expression pattern of α-l-fucosidase (*fucA* and *framefucA*) for *WT* and FFuc strains, respectively, was analyzed by using gene-specific primers FucFWD: 5′-TGCCAGATAGACCAGAACAC-3′ and FucREV5′-GCCCTATGATACGAAATGCC-3′ designed to amplify a 277 bp region of the α-l-fucosidase gene (SSO11867-SSO3060). Oligos were designed using the qPCR Probes Design Tool-GenScript (GenScript.com, Piscataway, NJ, USA). Real-time polymerase chain reactions (real-time PCR) were performed by using SYBR™ Green PCR Master Mix (Thermo Fisher, Waltham, MA, USA); each reaction (20 μL) mix contained 10 μL of Mastermix, 150 nM (final concentration) of each primer and 4 μL of cDNA (10 ng). PCR products were detected using Applied Biosystems 7300/7500/7500 Fast Real-Time PCR System (Applied Biosystems, Foster City, CA, USA) The program used was as follows: 10 min at 95 °C, 15 s at 95 °C and 1 min at 60 °C for 40 cycles. All samples were analyzed in triplicate. *fucA* and *framefucA* gene expression profiles were normalized against 16S transcript to correct for differences in the starting amount of RNA and in the efficiency of the reverse transcription reaction. Statistical significance was performed for all qRTPCR experiments using the two-tailed paired Student’s *t* test, and, for cold shock and different carbon sources growth experiments, also with ANOVA Dunnett’s test. Melting curve analyses of each PCR reaction were performed to assess specificity.

### 4.8. Optical Microscopy

Cell aliquots were collected during exponential phase and seeded on a slide glass and analyzed with an Olympus BX51 contrast phase microscope (Olympus corporation, Shinjuku, Tokyo, Japan), using a 100× lens.

### 4.9. Cell Lysates Preparation 

The cell pellets obtained after centrifugation were resuspended in lysis butter (20 mM potassium phosphate buffer pH 7.2, 150 mM NaCl, 0.1% Triton X 100) (1:5 *v*/*v*) and cell lysis was performed with 5 cycles of freeze and thaw. The supernatant was clarified by centrifugation at 12,000× *g* for 30 min, soluble fraction was separated, and protein concentration was measured with Bradford Protein Assay Kit (Bio-Rad, Hercules, CA, USA). Samples were kept frozen until use. 

### 4.10. Western Blot Analyses

*S. solfataricus* cells were lysed as described above and lysates were separated in 8% SDS-polyacrylamide gel and transferred to PVDF membranes (Merck-Millipore, Burlington, MA, USA). Filters were blocked for 3 h at RT in 5% (*w*/*v*) non-fat milk in PBS (Phosphate-buffered saline) 1X Tween-20 0.1% (TPBS).

For α-l-fucosidase, the filter was incubated with anti-FucA primary antibody (1:500, PRIMM srl., Milan, Italy). After several washings in TPBS, membranes were incubated with secondary antibody against mouse (1:50,000 PIERCE. Thermo Fisher, Waltham, MA, USA) linked to horseradish peroxidase, and signals were visualized by chemiluminescence (ECL, Amersham, Little Chalfont, UK).

For fucosylated glycoproteins the filter was incubated with the UEAI-HRP labelled antibody (0.1 mg/mL) for 2 h. After several washings in TPBS signals were visualized by chemiluminescence (ECL, Amersham, Little Chalfont, UK).

### 4.11. Alpha-L-Fucosidase Activity Assay

α-l-fucosidase activity assay was performed at 75 °C in 50 mM sodium phosphate buffer at pH 6.5, using 4-nitrophenyl-α-l-fucopyranoside (4NP-α-l-Fuc) substrate at the final concentration of 4 mM and different amount of *S. solfataricus WT* and FFuc cellular extracts. The reaction was blocked by adding 0.5 M Na_2_CO_3_ and the product, 4-nitrophenolate (4NP), was detected spectrophotometrically at 405 nm. The extinction coefficient used was = 18.2 mM^−1^cm^−1^. For all assays, spontaneous hydrolysis of the substrate was subtracted by using appropriate blank mixtures without cell lysates. Enzymatic activity assays have been performed in duplicate and reported as the mean ± SD. One unit (U) of enzymatic activity was defined as the amount of enzyme that released 1 umoL of 4NP per min at the conditions described. The units of enzymatic activity have been normalized for mg of total proteins in the cellular extracts.

## 5. Conclusions

Noticeably, α-l-fucosidases are extremely rare in Archaea and, up to now, *fucA* is the only gene known so far that is expressed by −1 PRF in this Domain of life. Several functional interrupted genes were identified in *S. solfataricus* [26] suggesting that in Archaea, more genes could be regulated by translational recoding, such as those in viral genomes or encoding for proteins with no enzymatic activity, but that have not yet been identified, possibly because of the difficulty of isolating and characterizing them. Under this point of view, the α-l-fucosidase activity is a useful molecular tool to study −1 PRF as it be easily assayed in vitro [17]. Here, the analysis of different growth conditions showed that cold shock and the presence of xyloglucan oligosaccharides increased up to 10-fold the mRNA abundance of *fucA*, while the full-length control gene showed mRNA levels similar and much less increased, respectively, if compared to standard growth conditions. We propose that the reason of the more abundant mRNA is due to the presence of ribosomes performing −1 PRF and thereby preventing its degradation. This may suggest that cold shock and xyloglucan oligosaccharides induce −1 PRF, but further studies, going beyond the aims of this work, are required.

It has been already postulated that the flexibility of the genetic code decoding is a trait selected during evolution to benefit microorganisms under certain conditions [27]. It is tempting to speculate that this regulation at translational level might be advantageous in extreme environments, which are often spots (e.g., for hydrothermal vents, solfataras, acidic/basic/salty ponds, etc.) located in places dominated by mild conditions. In these “extreme” sites, microbial communities might encounter sudden and reversible changes of the optimal growth conditions more frequently than microbes living in stable conditions. Therefore, translational recoding could be a way to maintain the expression of certain genes latent, and up- or down-regulate them under specific conditions. Approaches of system biology on the large amounts of available (meta) genomic data from extremophiles might open new avenues to the study of translational recoding in this domain of life [63].

## Data Availability

Data is contained within the article.

## Sample Availability

Samples are not available from the authors.
